# Mucopolysaccharidosis Type I and α-Mannosidosis—Phenotypically Comparable but Genetically Different: Diagnostic and Therapeutic Considerations

**DOI:** 10.3390/biomedicines13051199

**Published:** 2025-05-14

**Authors:** Marika Venezia, Martina Vinci, Paolo Colomba, Carmela Zizzo, Giovanni Duro, Marta Moschetti

**Affiliations:** Institute for Biomedical Research and Innovation (IRIB), National Research Council (CNR), 90146 Palermo, Italy; marika.venezia@irib.cnr.it (M.V.); martina.vinci@irib.cnr.it (M.V.); paolo.colomba@irib.cnr.it (P.C.); carmela.zizzo@irib.cnr.it (C.Z.); giovanni.duro@irib.cnr.it (G.D.)

**Keywords:** differential diagnosis, mucopolysaccharidosis, α-mannosidosis, epigenetics, artificial intelligence (AI)

## Abstract

Mucopolysaccharidosis type I (MPS-I) is an autosomal recessive, progressive, multisystem hereditary lysosomal storage disease (LSD), which is characterized by the gradual accumulation of dermatan sulphate (DS), heparan sulphate (HS), and glycosaminoglycans (GAGs) in all organs and tissues due to the deficiency of the enzyme α-L-hyduronidase. The multisystem clinical manifestations of varying severities of MPS-I are present in two forms—the “severe form of MPS I” (Hurler type) and the “attenuated form of MPS-I” (Hurler–Scheie or Scheie type). These forms represent the entire case history of the disease. The three phenotypes share common symptoms, including musculoskeletal abnormalities, facial dysmorphisms, hernias, short stature, finger stiffness, carpal tunnel syndrome, and corneal opacities. Abnormalities affecting the internal organs include hepatomegaly, splenomegaly, and valvulopathy. There is some evidence to suggest a similarity and overlap with the clinical symptoms of MPS-I, particularly in cases of another rare LSD that is autosomal and recessively inherited—l’α-mannosidosis. This disorder has been observed to result from a dysfunction of the corresponding α-mannosidase enzyme, which has been shown to lead to the accumulation of mannose-rich N-linked oligosaccharides. This review compares the phenotypic similarities and molecular differences between mucopolysaccharidosis type I (MPS-I) and α-mannosidosis. We review genotype–phenotype correlations, diagnostic difficulties, and the applicability of artificial intelligence for the assistance of differential diagnosis, with the goal of facilitating the earlier and more accurate diagnosis of these rare lysosomal storage diseases.

## 1. Introduction

MPS type I (MPS-I) is a rare autosomal recessive multisystem disorder caused by a deficiency of the lysosomal hydrolase α-L-hyduronidase (IDUA enzyme), which results in the accumulation of glycosaminoglycans (GAGs), dermatan sulphate (DS), and heparan sulphate (HS) in organs and tissues [1]. In consideration of the age of onset of initial symptoms, the rate of progression of the disease, as well as its predominant symptomatology, the condition is subdivided into three distinct phenotypes—Hurler (a more severe clinical phenotype), Hurler–Scheie (an intermediate clinical phenotype), and Scheie (a less severe clinical phenotype). However, it is interesting to note that recently, the classification has been reduced to two subgroups on the basis of neurological involvement—severe MPS-I (or Hurler syndrome) and attenuated MPS-I (or Hurler–Scheie syndrome) [2]. Hurler’s syndrome (MPS-IH), which is the most severe form of MPS-I, appears to affect the pediatric age group, with extremely low levels or an absence of IDUA enzyme activity thought to be the cause. It seems as though children appear normal at birth, with the first symptoms being displayed around the age of 6–8 months; the clinical signs that have been observed include corneal opacities, cardiac malformations, short stature, hernias, facial dysmorphisms, hirsutism, and organomegaly. The liver is the first organ to be affected, as a high level of GAGs has been found during the first months of life; then, other organs such as the brain are affected due to a pathological cascade of GAG accumulation [3]. The disease is believed to progress rapidly from the age of 3 years, and life expectancy is reduced in the first decade. The condition is characterized by a delay in psychomotor development and cognitive impairment. Many children with Hurler syndrome have a limited ability to speak due to cognitive decline, macroglossia (enlargement of the tongue), and reduced hearing. The skeletal system is characterized by progressive skeletal dysplasia (multiple dysostosis) involving all bones, as well as progressive arthropathy involving most joints.

In the first year of life, it appears that children with MPS-IH may be susceptible to certain deformities of the lower spine, as well as the potential involvement of the cardiac system. These include valvulopathy, which is a disease of the valves of the heart. If untreated, death can unfortunately occur within the first ten years of life due to cardiorespiratory failure. Other symptoms that may be present include hepatomegaly, splenomegaly, and hydrocephalus, i.e., the dilation of brain spaces with the accumulation of cerebrospinal fluid, which may occur in the second year of life. Children also manifest recurrent infections of the auditory system with recurrent otitis media and upper respiratory tract infections, often accompanied by profuse and persistent rhinorrhoea [1,4].

Scheie/Hurler–Scheie syndrome (IH/S) represents what is thought to be the attenuated form of MPS type I. This condition appears to manifest between the ages of 3 and 10, with progressive organ and system involvement. Mild hepatomegaly, relatively normal joint motion, mild dysostosis on skeletal radiographs, and mild corneal clouding are thought to lead to moderate somatic involvement, which is referable to the attenuated form. The coarseness of the facial features is less evident than in individuals with severe MPS-I, in whom a short neck, wide mouth, and square jaw are observed. At the joint level, the hands and shoulders are affected with carpal tunnel syndrome and reduced mobility; knee valgus and foot stiffness are other symptoms that can occur. Regarding cognitive development, some individuals have no neurological involvement, and their psychomotor development may be normal in early childhood, but learning difficulties and psychiatric manifestations may be present later in life. Depending on the severity and speed of disease progression, two cases may occur—death in the second or third decade, or a normal life characterized by significant disability due to progressive joint manifestations and cardiorespiratory disease. The progress of the disease is slower than in Hurler syndrome and survival is prolonged. Patients still have a progressive, disabling, and often fatal disease, with considerable disability but no cognitive impairment [1,5,6]. The main symptoms of MPS-I, such as dysmorphic features, dysostosis, and intellectual disability, are shared with the symptoms of α-mannosidosis, as was observed in a recent study by Wiesinger and colleagues. α-mannosidosis patients were found to have a similar phenotype to MPS-I patients [7] (Table 1).

α-mannosidosis is an ultra-rare autosomal recessive disorder caused by mutations in the *MAN2B1* gene, which encodes lysosomal alpha-mannosidase. This enzyme is responsible for cleaving α-mannosidic linkages during the breakdown of oligosaccharides [5]. Defective mannosidase activity prevents the degradation of glycoproteins, resulting in the lysosomal accumulation of mannose-rich oligosaccharide chains [7,8]. Individuals with α-mannosidosis have clinical symptoms that overlap with those of MPS I, including respiratory infections and skeletal changes [9]. There are three different forms—mild, moderate, and severe. The first form has a slow progression characterized by myopathy without skeletal atypia and is detected in the first decade of life. The second form is detected after the first decade and has a clinical profile identical to the first, except for the presence of skeletal abnormalities. In contrast, the severe form, which manifests in the first years of life, has a rapid progression and involves the central nervous system, leading to early death.

Systemic involvement is usually skeletal, characterized by bone disease such as asymptomatic osteopenia, focal lytic or sclerotic lesions and osteonecrosis, and craniofacial dysmorphism (macroglossia). In adulthood, cardiac complications and parenchymal lung disease may occur. The disease is characterized by gastrointestinal dysfunction and hepatosplenomegaly, hearing loss in early childhood, and frequent infections due to immunodeficiency. Sensorineural deafness, developmental delay/intellectual disability, ataxia, and spastic paraplegia are also seen. There are also rheumatological symptoms such as systemic lupus erythematosus [9]. Given the similarity of clinical presentations, it is essential to support clinicians in differentiating between mucopolysaccharidosis type I (MPS I) and α-mannosidosis—two disorders that despite having overlapping symptoms, are caused by mutations in distinct genes. A timely and accurate differential diagnosis is crucial to initiate appropriate treatment as early as possible. This review aims to provide a comprehensive overview of the currently available diagnostic procedures. The diagnostic approach typically begins with biochemical tests to assess enzymatic activity, followed by targeted genetic analyses. In addition to standard protocols, scientific research is increasingly focusing on the role of epigenetics to enhance diagnostic precision and enable earlier detection. Furthermore, the integration of artificial intelligence into diagnostic pathways represents a promising advancement for the early identification of rare diseases. Enzyme replacement therapy (ERT) with laronidase has demonstrated efficacy in reducing urinary glycosaminoglycan (GAG) levels, improving respiratory function, and decreasing hepatosplenomegaly. However, its effectiveness is limited in targeting skeletal, ocular, and central nervous system manifestations, mainly due to the restricted biodistribution of the enzyme to these tissues. Hematopoietic stem cell transplantation [HSCT] is considered the standard of care for patients with the severe form of MPS I (Hurler phenotype), especially when performed at an early disease stage. This intervention may help slow clinical progression and ameliorate several disease-related manifestations [10]. Gene therapy studies for MPS I are currently under development, aiming to directly address the underlying genetic defect responsible for the disease. Although preliminary results are encouraging, further research is required to assess the long-term safety and efficacy of this approach. For alpha-mannosidosis, ERT has proven effective in reducing urinary levels of mannose-rich oligosaccharides and improving certain clinical features. Nevertheless, its efficacy remains limited, particularly with respect to neurological symptoms, due to the enzyme’s poor penetration into the central nervous system. Hematopoietic stem cell transplantation (HSCT) has shown potential in slowing cognitive decline and improving hearing in some cases. However, its benefits on the brain and skeletal abnormalities are modest. The combined use of ERT before and during HSCT has yielded promising results in slowing disease progression and reducing oligosaccharide accumulation. This integrated therapeutic approach may contribute to improved long-term clinical stability [11].

## 2. α-Mannosidosis Diagnosis—Genotype–Phenotype Correlation Analysis in Patients with Clinical Manifestations Like MPS

MPS-I and α-mannosidosis have been shown to be caused by an enzyme deficit of α-L-hyduronidase and α-mannosidase, respectively, as a result of mutations in the corresponding genes (*IDUA* gene and *MAN2B1*). Mutations associated with the absence or deficiency of the enzymes of interest were analyzed to predict the corresponding disease phenotype. This analysis aims to strengthen diagnostic certainty and support genotype–phenotype correlation. Via the 3D reproduction of the enzymes α-L-hyduronidase and α-mannosidase, it was observed that missense mutations are those that cause disease with a more- or less-strong genotype–phenotype correlation.

**Figure 1:**
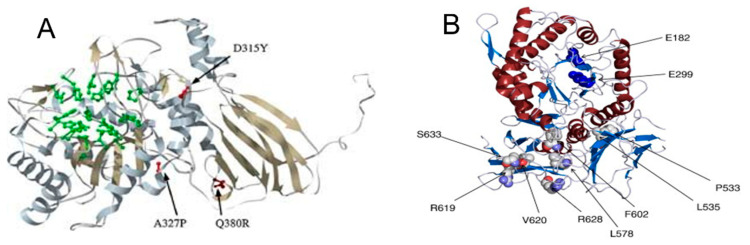
The mutations found in patients with Hurler syndrome (severe phenotype) in the core (**A**) and missense mutations (**B**).

With regard to MPS-I disease, a homology model of the IDUA protein was reproduced by one particular research group [12]. Based on the crystal structure of another protein of the glycosyl hydrolase 39 family—the beta-xylosidase from *Thermoanaerobacterium saccharolyticum*—the IDUA enzyme was constructed in 3D. This model showed that missense mutations, mainly located in the active site or in the hydrophobic core of the model protein, are associated with the severe phenotypes of MPS-1. In particular, the core mutations p.A327P and p.D315Y were found in patients with Hurler syndrome (severe phenotype) (Figure 1A). The p.Q380R mutation, which can be considered a less-severe mutation, is located on the surface of the protein [13]. Missense mutations such as p.V620F, p.R619G, and p.R628P are also associated with Hurler syndrome [14,15,16] (Figure 1B).

This region is also conserved to a lesser extent among mammalian IDUAs in the orthologous proteins of Danio rerio, Drosophila, Anopheles, and Tetraodon. Residues affected by missense mutations are highly conserved across species.

**Figure 2:**
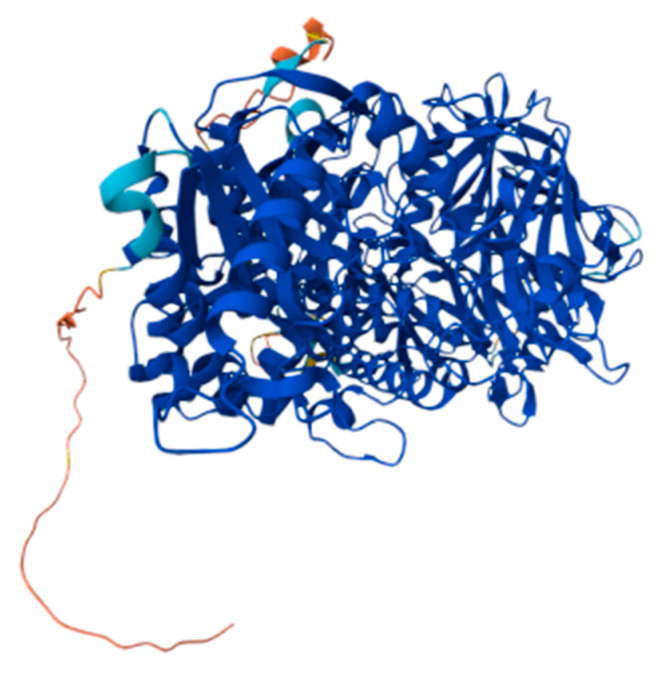
Lysosomal α-mannosidase structure (https://alphafold.ebi.ac.uk/entry/A0A2J7RN33, accessed on 13 April 2025).

Most surface-localized missense mutations result in the attenuated phenotype. The high degree of mutational heterogeneity in the IDUA enzyme is also found in α-mannosidosis. From OMIM (Online Mendelian Inheritance in Man), OMIA (Online Mendelian Inheritance in Animals), and previous studies, inherited mutations for α-mannosidosis have been identified, e.g., missense and nonsense insertions and deletions, as well as some splicing mutations [17,18,19]. A three-dimensional model was additionally developed to study alpha-mannosidosis, with a focus on missense mutations, which encompass substitutions within the protein sequence. A homology modelling approach was used to reconstruct the complete lysosomal α-D-mannosidase for humans, bovines, cats, and guinea pigs by mapping all mutations. It was observed that the feline 1748del4 mutation causes a severe genotype and a similarly lethal phenotype, resulting in the destruction of the enzyme structure, rendering it non-functional [20] (Figure 2).

Following a comprehensive analysis of known mutations, including non-splicing mutations known to cause α-mannosidosis, a close genotype–phenotype correlation was revealed. Javed Mohammed Khan and Shoba Ranganathan have identified five mutational hotspot regions within the *MAN2B1* gene, which are distributed throughout its sequence. This finding enhances the ability to predict the pathogenic potential of specific mutations and to pinpoint residues with a higher likelihood of being altered based on their genotypic location [20]. This is of crucial importance in genetic analysis, as it demonstrates a significantly higher rate of recombination or mutation in these regions when compared to other genomic regions. The identification of hot spot regions is critical for the comprehension of the pathogenetic mechanisms, as they can significantly influence the severity and phenotypic variability of the disease. This phenomenon can therefore condition the onset of specific clinical conditions based on gene localization. The previously mentioned authors extrapolated mutations from a range of organisms (humans, cows, guinea pigs, and cats) and their proximity to the active site of the enzyme. They succeeded in highlighting the effect of the disease mutations on the structure of the proteins, which forms the basis for understanding the molecular determinants for phenotypic variations [20]. The present study proposes that given the essential nature of lysosomal α-mannosidase and the fact that all the mutations observed affect its function, this disease could be tackled by gene therapy for inherited diseases rather than by the development of drugs or inhibitors. The observation of hotspot mutations can therefore assist clinicians in the development of a personalized treatment plan, during which the disease response and potential development of drug resistance can be monitored.

## 3. Misdiagnosis of α-Mannosidosis: A Case Study Comparing MPS and α-Mannosidosis from a Clinician’s Perspective

Despite the phenotypical similarity, MPS-I and α-mannosidosis exhibit distinct genetic distinctions. LSDs demonstrate clinical similarities in numerous respects; however, their genetic underpinnings differ. This phenomenon was elucidated through a case study conducted by Öckerman [21], a Swedish physician specializing in pediatrics, as demonstrated in 1967. The study involved an investigation of the case of a pediatric patient who, at the age of four, presented a phenotype that exhibited similarity to Hurler’s syndrome [21]. The patient succumbed early to pneumonia at the age of 4, and large quantities of oligosaccharide material with a prevalence of mannose were observed in his tissues. This observation led to the term ‘mannosidosis’ being used as a nickname for this LSD. A decade later, in 1977, Loeb described an atypical form of mucopolysaccharidosis that was later found to be α-mannosidosis [22]. Recent studies have focused on the need to consider this rare disease from a clinical and diagnostic perspective, particularly in patients suspected of having MPS-I. Specifically in 2020, Thomas Wiesinger and colleagues conducted a pilot study analyzing 1010 dried blood samples of individuals suspected of having MPS-I from the Middle East and Europe. Following the execution of enzymatic testing, samples that exhibited a positive result for MPS-I underwent NGS, a process which confirmed the diagnosis for 158 samples, including 52 MPS-I, 2 MPS II, 0 MPS IIIb, 71 MPS IVa, 33 MPS VI, and 0 MPS VII. For the remaining 835 samples that exhibited MPS-like symptoms but were negative for MPS, further biochemical analyses were conducted to assess α-mannosidase activity. Samples with a reduced enzyme activity (<1st percentile) underwent genetic confirmation, leading to the identification of four individuals with a final diagnosis of α-mannosidosis, indicating an incidence of 1 in 253 individuals with an MPS-like phenotype. These data underscore the underdiagnosis of α-mannosidosis, suggesting a significantly higher frequency of 1 in 500,000 in Europe and the Middle East regions [7]. The findings of this study indicate that α-mannosidosis should be considered in the context of a differential diagnosis in individuals exhibiting signs and symptoms that are consistent with an MPS-like phenotype. In recent years, the scientific community has emphasized the need to consider mucopolysaccharidosis (MPS) and α-mannosidosis concurrently in diagnostic workflows in order to enhance the accuracy and timeliness of differential diagnosis. Supporting this perspective, a 2024 publication reported a study involving 250 patients with suspected MPS but no confirmed diagnosis, which aimed to investigate the potential presence of α-mannosidosis. Enzymatic activity analysis revealed abnormalities in 53 samples, which were subsequently subjected to *MAN2B1* gene sequencing using the Sanger method. In three families, the pathogenic p.Ser899Ter variant was identified, resulting in a truncated protein with no residual enzymatic activity. These findings suggest that α-mannosidosis is frequently underdiagnosed due to both significant phenotypic overlap with MPS and limited clinical awareness of this ultra-rare condition [23]. In light of these study hypotheses, it is considered essential to perform specific biochemical tests in parallel to assess enzymatic activity for mucopolysaccharidosis type I (MPS I) and α-mannosidosis in order to allow clinicians to evaluate both conditions simultaneously. This strategy enables timely differential assessment by the clinician, facilitating the early identification of clinical presentations that are consistent with one of the two lysosomal disorders. If one of the two enzymatic assays reveals an activity below the reference range, second-level investigations through molecular analysis are recommended, in accordance with the approach described by Wiesinger et al. [7]. Specifically, the application of Sanger sequencing to the *IDUA* gene (encoding α-L-iduronidase) and the *MAN2B1* gene (encoding lysosomal α-mannosidase) represents a targeted approach for detecting pathogenic variants, enabling a definitive genetic diagnosis. This combination of biochemical and genetic analyses enhances diagnostic accuracy and supports the early initiation of an appropriate therapeutic pathway.

## 4. The Roles of Epigenetics in MPS-I and α-Mannosidosis

Lysosomal storage diseases (LSDs) are a group of monogenic disorders that can range from neonatal onset to symptoms developing in late adulthood. This suggests that a significant role is played by epigenetic factors in phenotypic variability. The broad spectrum of associated phenotypes is a hallmark of these conditions, and the clinical heterogeneity observed in such disorders gives rise to the consideration of epigenetics as a potential factor in disease progression. In his study, Vargas-López investigated epigenetic mechanisms in mucopolysaccharidosis IIIB (MPS IIIB) and IVA (MPS IVA), analyzing DNA methylation and histone modifications (H3K14 acetylation and H3K9 trimethylation) in fibroblasts from patients and healthy controls. The results revealed global DNA hypomethylation in both disorders, accompanied by increased histone acetylation in a donor-dependent manner, which is indicative of a more open chromatin state. A reduction in H3K9 trimethylation clustering was observed only in MPS IIIB cells, suggesting limited heterochromatin alterations [24]. The most common epigenetic mechanisms include DNA methylation, histone modifications, and microRNAs (miRNAs), which play a role in gene regulation, phenotypic variation, and therapy. These processes, mediated by specific enzymes, influence gene expression without altering the DNA sequence and are essential for development and physiological balance. However, abnormalities in these regulatory mechanisms contribute to the onset of numerous diseases. This awareness has fostered the development of therapies targeting altered epigenetic enzymes. Over the past two decades, numerous small-molecule compounds—including inhibitors of DNA methyltransferases, histone deacetylases, and isocitrate dehydrogenase—have been studied and employed, particularly in the field of oncology. One example is this review, which examines the role of epigenetics in both normal and pathological conditions, highlighting major advances in the development and clinical application of epigenetic agents, including inhibitors, agonists, and multitarget molecules, identifying current challenges and future research opportunities [25]. These mechanisms have also been analyzed for some lysosomal storage diseases, including Pompe, Fabry, and Gaucher disease [26,27,28]. The persistent variability of clinical manifestations and the absence of a close genotype–phenotype correlation in MPS-I and α-mannosidosis diseases suggest that epigenetic studies may be useful in elucidating the mechanisms underlying these complex disorders.

The methylation of cytosine-phosphate-guanine (CpG) dinucleotides, particularly in gene promoter regions and other regulatory elements, has been shown to directly inhibit the binding of transcription factors [29,30]. This process has been observed to result in the recruitment of CpG methyl binding domain (MBD) proteins, histone deacetylases, and other factors implicated in the alteration of chromatin architecture. The potential repercussions of this phenomenon could be detrimental, with a plausible outcome including a reduction in the expression of the α-mannosidase and IDUA enzymes, thus promoting the progression of the disease.

Another class of epigenetic mechanisms that functions by means of directly restructuring the complex formed between DNA and/or chromatin includes histone modifications, with acetylation being a notable example. This is a more studied modification, which controls the binding of chromatin remodelling factors by influencing gene expression at the transcriptional level. Another factor that may be implicated is microRNAs (miRNAs), which are small non-coding RNAs that regulate post-transcriptional gene expression [31]. In light of this study, researchers are striving to better understand cellular processes and human diseases, while also uncovering the influence of environmental factors on phenotypes. These mechanisms are particularly relevant in explaining the clinical heterogeneity of monogenic disorders, such as lysosomal storage diseases (LSDs), which exhibit marked phenotypic variability and often weak genotype–phenotype correlations. The review by Hassan is a notable example, exploring the main epigenetic mechanisms and their role in phenotypic variability and therapeutic prospects, with a focus on three representative LSDs—Gaucher disease, Fabry disease, and Niemann–Pick type C—to encourage interest in epigenetics as a key to interpreting the biological complexity of these rare disorders [32].

The identification of specific miRNA signatures in different individuals presenting with clinical signs and symptoms of MPS or alpha-mannosidosis could contribute to future research aimed at understanding the underlying molecular mechanisms. Circulating extracellular miRNAs, found in the bloodstream within exosomes or in complexes with proteins and lipoproteins, could serve as unique signatures associated with diagnosis. According to Nasrin Khan et al., the capillary electrophoresis–mass spectrometry (CE-MS) method enables a multiplex and direct analysis of miRNAs from biological samples [33]. In particular, with regard to the response to enzyme replacement therapy (ERT), it has been observed that microRNAs (miRNAs) can regulate post-treatment. In the context of therapeutic interventions, the prevailing recommendation is to identify the epigenetic mechanisms that influence both diseases. This identification process is intended to facilitate the development of specific treatments that have the potential to enhance the efficacy of ERT [26]. Studies on epigenetics would be of paramount importance with regard to disease progression and therapy, as it is believed to have influences not only on disease progression but also on response to treatment.

## 5. Could AI Be Useful to Distinguish MPS-I and α-Mannosidosis?

Artificial intelligence (AI) is a tool that is currently gaining ground with excellent results and has already been adopted by researchers in several types of lysosomal storage diseases. In particular, it could be useful in the differential diagnosis of rare diseases such as mucopolysaccharidosis type I (MPS-I) and α-mannosidosis. These two inherited lysosomal diseases are characterized by common clinical symptoms such as cognitive impairment, hearing loss/deafness, dysostosis, facial dysmorphism, and multi-organ dysfunction. As AI has shown significant potential in processing and analyzing large amounts of clinical and genomic data through algorithms that can analyze subtle phenotypic differences, it could assist the clinician in identifying the possible disease. In recent years, artificial intelligence (AI) has seen increasing use in healthcare, demonstrating significant potential in improving the efficiency of diagnosis and treatment. Technologies such as machine learning enable the analysis of large datasets, the identification of patterns, and the generation of predictions that are useful for medical diagnosis and therapy personalization. In particular, AI can overcome some of the limitations that are typical of rare diseases, helping to optimize traditional clinical trials and reduce pharmaceutical research costs. Recent advancements also allow models to be trained on large datasets, which are subsequently adapted to the smaller datasets typical of rare diseases. Wojtara et al. examined the latest developments in AI and its applications in the diagnosis and treatment of rare diseases [34]. Another recent study has shown that AI can significantly reduce diagnostic time, enabling early diagnosis in a shorter timeframe, which is crucial for more timely and targeted therapeutic interventions [26].

Once a hypothetical cohort of patients has been identified using AI, traditional differential diagnosis will allow us to carry out an in-depth analysis of clinical data, with enzymatic tests being confirmed by genetic tests. However, the limited availability of data represents a significant obstacle to diagnosis, even for professionals with extensive clinical experience, contributing to the so-called ‘diagnostic odyssey’ that many patients face. To address this challenge, it may be necessary to develop innovative and automated tools to support clinicians. Machine learning emerges as a technology with high potential in this field, although its application in relation to rare diseases raises complex methodological, technological, and ethical issues. In this context, Decherchi offers a critical analysis of the interaction between rare diseases and machine learning techniques, highlighting key problem areas and providing methodological perspectives that could help generate new knowledge for the benefit of patients [35].

In conclusion, the implementation of AI in the diagnostic process represents a crucial development in modern medicine, providing advanced tools for the differential diagnosis between MPS-I and α-mannosidosis. This synergy between clinical expertise and advanced technology not only improves the quality of care but also opens up new perspectives for the management of rare diseases.

## 6. Discussion and Conclusions

Among the different disease categories, rare diseases are the most complex to identify, diagnose, and treat. In particular, lysosomal storage diseases (LSDs) include MPS-I and α-mannosidosis. Both share a deficiency of lysosomal enzymes, such as α-L-hyduronidase in MPS-I and α-mannosidase in α-mannosidosis, due to mutations in the genes encoding these enzymes (*IDUA* and *MAN2B1* genes, respectively). Patients suffering from these diseases have been found to display similar phenotypic characteristics and manifestations. However, due to an apparent absence of a definitive correlation between the symptoms exhibited and the corresponding disease, it is imperative to undertake a thorough investigation of clinical cases that present with symptoms analogous to those observed in MPS-I. As a first investigative test, we start with biochemical tests; then, in the case of patients with MPS-I, we proceed with the integration of the measurement of urinary GAG levels. People with MPS-I can excrete excess GAGs in the urine, and proportions of GAG types are sometimes found in the urine compared to healthy people of the same age.

Despite the absence of a specific screening test, it is recommended that a comprehensive urine profile be conducted, encompassing a range of quantitative and qualitative analyses. It is imperative that the urine sample does not exceed the acceptable limits of dilution (specific gravity < 1.015), as this may potentially result in a false negative outcome [36]. Urine GAG analysis can therefore suggest the most likely type of MPS, but genetic testing is required to confirm the suspected disease. While for MPS-I, this procedure is performed by physicians, for α-mannosidosis, a higher level of involvement and attention is required to avoid the misdiagnosis of this ultra-rare disease.

**Figure 3:**
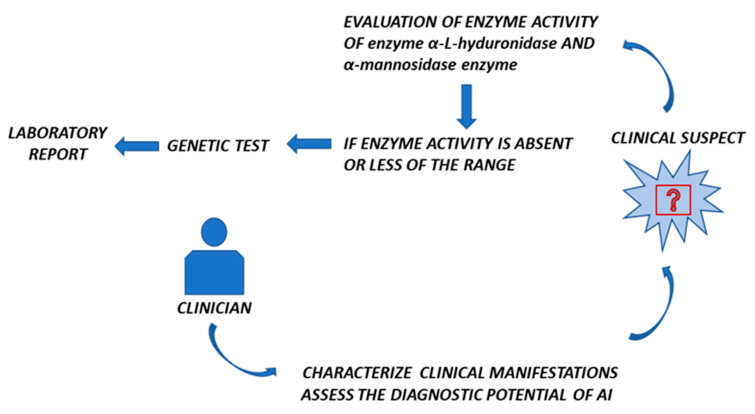
Visual decision for differential diagnosis.

In conclusion, MPS-I and α-mannosidosis are so similar symptomatically that this review aims to suggest that the clinician consider and include genetic testing for α-mannosidosis in patients who present with a phenotype similar to that of MPS-I, accompanied by negative enzymatic results for IDUA [7]. In addition to the classic protocols performed by the laboratory, with enzymatic assays and genetic tests, it would be advisable to incorporate diagnostic protocols using artificial intelligence in order to reduce the time required for early and differential diagnosis and to have a targeted and customized therapy in the shortest possible time (Figure 3). Therefore, we reiterate the importance of testing for α-mannosidase in all patients suspected of having MPS-I but with a negative result for the diagnosis of MPS-I.

Therefore, the main challenges in the study and management of rare diseases include the limited availability of epidemiological data, the fragmentation of research efforts, and the scarcity of large-scale clinical trials. The contribution of well-designed epidemiological studies, together with the introduction of artificial intelligence-based tools, represents a promising perspective to support clinicians in the early diagnostic process and in raising clinical suspicion.

## Figures and Tables

**Table 1:** MPS-I and alpha-mannosidosis disease phenotypes. Comparative table between mucopolysaccharidosis type I (MPS I) and α-mannosidosis, highlighting key phenotypic, genetic, and clinical differences relevant for differential diagnosis. Distinctive features of these lysosomal storage disorders are summarized, including type of hearing loss, presence of corneal opacity, immunodeficiency, and skeletal abnormalities.

MPS-I		α-Mannosidosis	
**MPS IH** Hurler syndrome (severe form)	**From 6 to 8 months** Corneal opacity Facial dysmorphisms Hirsutism Organomegaly Cardiac malformations Short stature Hernias **From 1 to 3 years** Gibbus deformities of the spine Valvulopathy Cognitive decline Macroglossia Hypoacusis Multiple dysostosis Progressive arthropathy Hepatomegaly Splenomegaly Hydrocephalus Recurrent otitis media Rhinorrhoea	**Mild form**	**Within 10 years** Slow progressin characterized by myopathy
		**Moderate**	**After 10 years** Slow progression characterized by myopathy and skeletal abnormalities
		**Severe**	**Early life** Rapid progression with central nervous system involvement leading to early death
		**Main symptoms** Immunodeficiency Asymptomatic osteopenia Focal lytic or sclerotic lesions and osteonecrosis Craniofacial dysmorphisms Macroglossia Cardiac complications Parenchymal lung disease Gastrointestinal dysfunction Hepatosplenomegaly Hearing loss in early childhood Frequent infections Sensorineural deafness Developmental delay Intellectual disability Ataxia Spastic paraplegia Systemic lupus erythematosus	
**MPS IH/S** Hurler–Scheie (mild form)	**From 3 to 10 years** Slight corneal opacity Mild facial dysmorphisms Mild hepatomegaly Mild dysostosis Carpal tunnel syndrome Knee valgism Foot stiffness Possible learning difficulties Psychiatric manifestations		
